# Fosfomycin Susceptibility Patterns Among Uropathogens and Clinical Predictors of Resistance: A Cross-Sectional Study at a Tertiary Care Centre in Northern India

**DOI:** 10.7759/cureus.108724

**Published:** 2026-05-12

**Authors:** Gaurav Badoni, Vanya Singh, Ranjana Rohilla, Tanisha Sharma, Vikas K Panwar, Balram J Omar, Pratima Gupta

**Affiliations:** 1 Microbiology, All India Institute of Medical Sciences, Rishikesh, Rishikesh, IND; 2 Urology, All India Institute of Medical Sciences, Rishikesh, Rishikesh, IND

**Keywords:** bacterial uropathogens, esbl uti, fosfomycin sensitivity, uncomplicated urinary tract infection, uti treatment

## Abstract

Introduction: Urinary tract infections (UTIs) represent a significant global health concern, with *Escherichia coli* (*E. coli*) and *Klebsiella pneumoniae *(*K. pneumoniae*) as predominant uropathogens. Rising multidrug resistance, including extended-spectrum β-lactamase (ESBL)-producing strains, has renewed interest in fosfomycin as an oral therapeutic option. This study evaluated the fosfomycin susceptibility pattern among uropathogenic *E. coli *and *K. pneumoniae *in northern India.

Method: This cross-sectional study was conducted at All India Institute of Medical Sciences (AIIMS), Rishikesh, from January to May 2025. Antimicrobial susceptibility testing (AST) of 407 culture-positive UTI isolates was performed using the VITEK-2 compact system (GN-235 cards) (bioMérieux SA, Marcy-l’Étoile, France), with results interpreted as per Clinical and Laboratory Standards Institute (CLSI) guidelines. Multivariable logistic regression and receiver operating characteristic (ROC) curve analysis identified independent predictors of fosfomycin resistance.

Results: In this study, the participants were predominantly male, 242 (55.37%), with a median age of 50 years. *E. coli *accounted for 303 (69.3%) isolates and demonstrated high fosfomycin susceptibility, 302 (86%). On the other hand, *K. pneumoniae*, 104 (26%), exhibited alarmingly high resistance, 53 (98%). Bacterial species was the sole independent predictor of resistance, with *K. pneumoniae *being over 300 times more likely to be resistant than *E. coli *(OR = 322; 95% CI: 68.0-5765; p < 0.001). Age and sex were non-significant. The model showed excellent discrimination (AUC = 0.925).

Conclusion: Fosfomycin showed excellent activity against *E. coli*, supporting its role as first-line therapy for uncomplicated UTI. However, the high resistance rate in *K. pneumoniae *strongly cautions against empirical fosfomycin use when this pathogen is suspected, reinforcing culture-guided prescribing practices.

## Introduction

Urinary tract infections (UTIs) are one of the leading causes of illness worldwide, affecting people of all ages and caused by a variety of microorganisms. According to published data, around 12% of males and children, as well as 50% of all females, will suffer from UTI during their lifetime [1]. Asymptomatic bacteriuria, pyelonephritis, and chronic or recurrent disease are also clinical presentations of UTI, in addition to cystitis, which is the most frequent. The Infectious Diseases Society of America (IDSA) updated the guidelines for UTI as complicated and uncomplicated UTI in 2025 [2], in which complicated UTI occurs when infection goes beyond the bladder in both men and women, whereas in uncomplicated UTI, infection remains within the bladder in afebrile patients [3].

Uncomplicated UTI develops when a person's urinary system is free of abnormalities. On the other hand, complicated UTI develops in people who are predisposed to illnesses such as kidney disease, pregnancy, urinary tract blockage, nerve damage, immunocompromised status, stones, and the use of medical devices such as catheters [4]. *E. coli* is the leading microorganism causing UTI, with increasing antibiotic resistance creating serious concern for public health. According to published data, resistance to regularly prescribed antibiotics such as ciprofloxacin, amoxicillin, and trimethoprim/sulfamethoxazole is increasing. The prevalence of multidrug-resistant (MDR) and extended-spectrum β-lactamase (ESBL)-producing uropathogenic isolates continues to increase, underscoring fosfomycin as a promising oral therapeutic option for managing such infections [5].

Fosfomycin disrupts peptidoglycan precursor synthesis, specifically UDP-N-acetylmuramic acid, through a covalent bond with the Cys-115 residue in the enzyme's active site [6]. Its distinct mechanism, independent of other antibiotics, minimises the potential for cross-resistance with conventional agents. While resistance in uropathogenic *E. coli* varies by geographic region and remains generally low worldwide, supporting its utility for UTI therapy, a recent rise in resistance has surfaced, especially among ESBL-producing strains in some parts of the world. Studies demonstrate strong susceptibility of ESBL-producing *E. coli* to fosfomycin; however, resistance emerges as its use increases due to the high number of prescriptions. Documented studies from northern India on the prevalence of fosfomycin resistance in *E. coli* from UTI patients highlight the need for awareness programmes and community-level studies to identify such issues at regular intervals. The wild-type *K. pneumoniae* population demonstrates fosfomycin MIC values at or below 64 mg/L, yet in vitro activity is substantially undermined by hetero-resistance and diverse resistance mechanisms such as the intrinsic fosA gene, transporter gene defects, regulatory mutations, and target-site modifications [7]. It is acknowledged that current Clinical and Laboratory Standards Institute (CLSI) M100 guidelines (2024 edition) [8] do not provide validated interpretive breakpoints for oral fosfomycin against *K. pneumoniae*, and the IDSA 2024 AMR Guidance [9] advises against fosfomycin use for *K. pneumoniae* UTI, given the risk of clinical failure driven by the fosA gene. However, documented data on the high prevalence of fosfomycin in *K. pneumoniae*, guideline recommendations that advice against fosfomycin use in *K. pneumoniae* UTI, and guidelines to guide clinicians in this region to avoid empirical fosfomycin prescribing when *K. pneumoniae* infection is suspected remain limited.

The present study focused on the prevalence of fosfomycin-resistant *E. coli* in UTI-suspected patients coming from different regions of northern India. In addition, this study also analyses the proportion of the second most common causative agent of UTI, i.e., fosfomycin-resistant *Klebsiella *strains, although current CLSI guidelines do not provide fosfomycin breakpoints against *Klebsiella*.

## Materials and methods

Patient data collection

The present study was conducted at All India Institute of Medical Sciences, Rishikesh, from January 1, 2025, to May 31, 2025. Data were collected from patients based on demographic and clinical information, including age, gender, inpatient/outpatient status, history of previous UTIs, comorbidities (e.g., diabetes, catheterisation, renal conditions), and travel history. All genders and age groups were included in the study, whose demographic history, antimicrobial susceptibility, and clinical information were available. Non-*E. coli *and non-*Klebsiella* isolates, non-urinary infections, duplicate or contaminated isolates, incomplete documentation, and withdrawal of consent were excluded from the study.

Sample collection and processing

The study population comprised patients presenting with clinically suspected urinary tract infections, including both inpatient and outpatient settings, from whom urine samples were received for routine microbiological analysis. Patients of all genders and age groups, with no prior history of antibiotic use within three months and willing to provide informed consent, were enrolled. Mid-stream urine was collected in sterile universal containers following standard clinical procedures and sent to the laboratory immediately. While all patients suspected of having a UTI were initially screened, the study exclusively utilised samples that yielded positive cultures for *E. coli *and *Klebsiella*. The samples were routinely streaked onto Cystine-Lactose-Electrolyte-Deficient (CLED) agar and maintained under aerobic conditions at 37°C for a period of 18 to 24 hours. Colony growth and bacterial density were evaluated according to standard protocol. To confirm the identity of the isolates, the VITEK-2 automated system (bioMérieux SA, Marcy-l’Étoile, France) was used.

Sample size

The sample size was calculated using the following formula

$n = \frac{Z&sup2; &times; P(1-P)}{d&sup2;}$

with a prevalence (P) of 6.9% obtained by Mohapatra et al. (2022) [10], a 95% confidence level (Z = 1.96), and a 5% margin of error (d = 0.05), the calculated minimum sample size was 99. However, the authors used a total population sampling approach to maximise the study power, enrolling all participants who met the inclusion criteria.

Susceptibility analysis

Following the manufacturer's protocol, antimicrobial susceptibility testing (AST) was conducted via the VITEK-2 Compact system (bioMérieux, France) utilising GN-235 cards. The isolates were tested against a wide range of antibiotics, including ampicillin, amoxicillin/clavulanic acid, ticarcillin, piperacillin/tazobactam, cefoxitin, cefixime, ceftazidime, ceftriaxone, ertapenem, amikacin, gentamicin, nalidixic acid, ciprofloxacin, norfloxacin, ofloxacin, fosfomycin, nitrofurantoin, and trimethoprim/sulfamethoxazole. Result interpretation was standardised based on the latest CLSI 2025 guidelines [8]. Quality control tests were performed as per standard laboratory practices using recommended control strains.

Statistical analysis

All statistical analyses were carried out using R Studio (version 4.3.x; R Core Team, 2024). Continuous variables were reported as median (IQR) and compared using the Wilcoxon rank-sum test, while categorical variables were presented as frequencies and percentages and compared using Pearson's χ² test or Fisher's exact test, respectively. Multivariable binary logistic regression was used to identify independent predictors of fosfomycin resistance, which were presented as aOR with 95% CI. Model calibration was evaluated using the Hosmer-Lemeshow test and a bootstrap calibration curve (B = 200). AUROC (area under the receiver operating characteristic curve) was used to assess discriminative performance, with Youden's J index determining the optimal threshold. To determine the most influential variables, a Random Forest algorithm consisting of 500 trees was utilised, measuring importance through the average reduction in Gini impurity. These findings informed the creation of a nomogram designed to estimate personalised resistance risks. All statistical analyses were held to a significance threshold of p < 0.05 (two-tailed).

## Results

Demographic distribution

**Figure 1:**
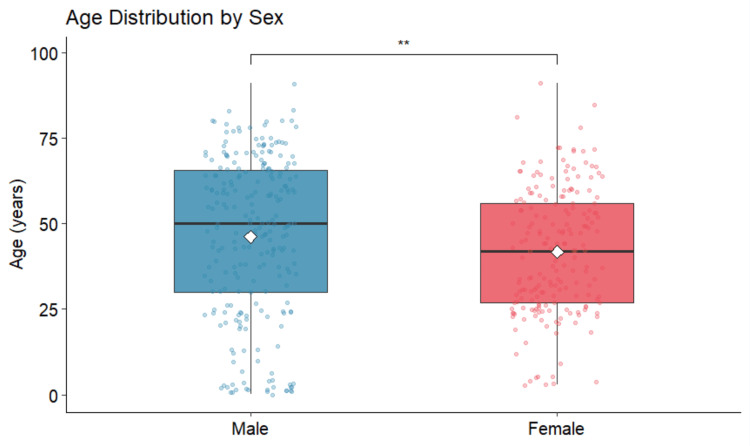
Age distribution of UTI patients. ** denotes a statistically significant difference between groups (p<0.01). UTI: urinary tract infection.

A total of 437 patients tested positive for UTI from January 2025 to May 2025, of whom 242 (55.37%) were males and 195 (44.6%) were females, with mean ages of 46 ± 23 years (50, IQR 30-66) and 42 ± 18 years (42, IQR 27-56) for males and females, respectively, and an overall median age of 47 years (IQR: 27-61) (Figure 1). This age disparity was further reflected in the categorical distribution across age groups (p < 0.001). Female patients, 82 (42%), predominated in the adult population compared with male patients, 53 (22%), while older males, 88 (36%), were greater in number (Table 1).

**Figure 2:**
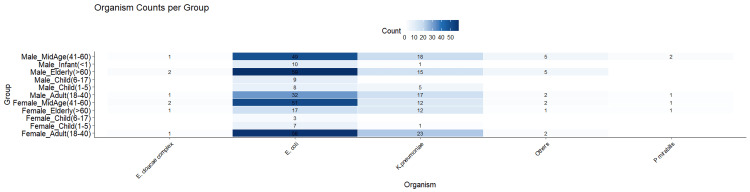
Heatmap illustrate showing predominant microbial species present in the UTI patients. UTI: urinary tract infection.

Regarding the diverse microbial profile, *E. coli*, 303 (69.33%), was the most frequent pathogen identified among the total patients, followed by *K. pneumoniae*, 104 (23.79%), *Enterobacter cloacae* complex, 8 (1.83%), *Proteus mirabilis*, 5 (1.14%), and some other species (*C. freundii*, *C. koseri*, *E. asburiae*, *K. cryocrescens*, *K. intermedia*, *M. morganii*, *P. agglomerans*, *R. planticola*, *S. fonticola*, *S. marcescens*, *Y. kristensenii*), 17 (3.89%), all of which showed a balanced distribution across genders (Table 1). The heatmap showed that *E. coli* was the predominant uropathogen across all age-sex subgroups, with the highest burden observed in adults (18-40 years) of both genders, followed by *K. pneumoniae*, which was more common in the middle-aged (41-60 years) population and elderly males (>60 years). *P. mirabilis *and *E. cloacae* complex were isolated in smaller numbers in older age groups, consistent with their association with complicated UTIs (Figure 2). 

**Table 1:** Patient characteristics. Data are presented as mean ± SD/median (Q1–Q3) for continuous variables and as n (%) for categorical variables. p-value: Wilcoxon rank sum test; Pearson’s chi-squared test.

Characteristics	Overall, N = 437	Male, N = 242	Female, N = 195	p-value
Age (mean ± SD)	44 ± 21	46 ± 23	42 ± 18	0.005
Median	47, IQR 27–61	50, IQR 30–66	42, IQR 27–56	-
Age group	-	-	-	<0.001
Neonate/infant (<1)	1 (0.22%)	1 (0.41%)	0 (0%)	
Child (1–5)	28 (6.40%)	23 (9.50%)	5 (2.56%)	
Child/adolescent (6–17)	15 (3.43%)	9 (3.71%)	6 (3.07%)	
Adult (18–40)	135 (30.89%)	53 (21.90%)	82 (42.05%)	
Middle-aged (41–60)	132 (30.20%)	68 (28.09%)	64 (32.82%)	
Elderly (>60)	126 (28.83%)	88 (36.36%)	38 (19.48%)	
Organism	-	-	-	0.7
E. coli	303 (69.33%)	167 (69.00%)	136 (69.74%)	
K. pneumoniae	104 (23.79%)	56 (23.14%)	48 (24.61%)	
*E. cloacae* complex	8 (1.83%)	4 (1.65%)	4 (2.05%)	
P. mirabilis	5 (1.14%)	3 (1.23%)	2 (1.02%)	
Others	17 (3.89%)	12 (4.95%)	5 (2.56%)	

ESBL resistance (*E. coli, Klebsiella*)

Analysis of patient characteristics by ESBL status revealed distinct patterns between *E. coli *and *Klebsiella *infections. In the *E. coli *group (303, 69.33%), age was not a determining factor for ESBL presence, with a mean age of 43.69 ± 21.87 years and no significant difference between the ESBL-absent and ESBL-present groups (p > 0.005). Similarly, the distribution across age groups showed no statistical significance (p > 0.005). However, gender was a highly significant predictor of *E. coli *ESBL status (p < 0.001), with males disproportionately represented in the ESBL-positive group (70, 70.70%) compared to females (29, 29.29%) (Table 2).

**Table 2:** Patient characteristics by E. coli ESBL status. Data are presented as Mean ± SD/median (Q1–Q3) for continuous variables and as n (%) for categorical variables. p-value: Wilcoxon rank sum test; Pearson’s chi-squared test. ESBL: extended-spectrum β-lactamase.

Variable	Overall, N = 303	Absent, N = 204	Present, N = 99	p-value
Age (mean ± SD)	43.69 ± 21.87	43.75 ± 21.44	43.58 ± 22.86	0.936
Median	45.00 (27.00-61.00)	44.50 (27.50-60.00)	46.00 (26.00-63.00)	-
Sex	-	-	-	-
Female	136 (44.88%)	107 (52.45%)	29 (29.29%)	<0.001
Male	167 (55.11%)	97 (47.54%)	70 (70.70%)	
Age group	-	-	-	0.814
0-14	36 (11.88%)	22 (11.78%)	14 (14.14%)	-
15-30	56 (18.48%)	38 (18.62%)	18 (18.18%)	-
31-45	60 (19.80%)	43 (21.07%)	17 (17.17%)	-
46-60	75 (24.75%)	52 (25.49%)	23 (23.23%)	-
>60	76 (25.08%)	49 (24.01%)	27 (27.27%)	-

In contrast, *Klebsiella *(104, 23.79%) demonstrated no statistically significant associations between ESBL status and any patient variables. The mean age was 43.47 ± 20.66 years, and no significant variation was observed between the ESBL-absent and ESBL-present groups (p > 0.005) or across age categories (p > 0.005). Unlike the *E. coli *findings, gender did not correlate with *Klebsiella *ESBL status (p > 0.005), with nearly identical gender distributions observed in both the presence and absence of the enzyme. These results indicate that while male sex is a significant risk factor for ESBL-producing *E. coli*, it does not appear to influence ESBL status in *Klebsiella *infections within this study population (Table 3).

**Table 3:** Patient characteristics by Klebsiella ESBL status. Data are presented as mean ± SD/median (Q1–Q3) for continuous variables and as n (%) for categorical variables. p-value: Wilcoxon rank sum test; Pearson’s chi-squared test. ESBL: extended-spectrum β-lactamase.

Variable	Overall, N = 104	Absent, N = 44	Present, N = 60	p-value
Age (mean ± SD)	43.47 ± 20.66	41.84 ± 21.59	44.67 ± 20.05 / Median: 46.00 (28.50-62.00)	0.5
Median	45.50 (IQR 26.50-61.00)	44.50 (IQR 24.00-60.00)	46.00 (IQR 28.50-62.00)	
Sex	-	-	-	>0.999
Female	48 (46.15%)	20 (45.45%)	28 (46.66%)	-
Male	56 (53.84%)	24 (54.54%)	32 (53.33%)	-
Age group	-	-	-	0.94
0-14	7 (6.73%)	4 (9.09%)	3 (5.0%)	-
15-30	25 (23.07%)	11 (25.0%)	14 (23.33%)	-
31-45	20 (19.23%)	8 (18.18%)	12 (20.0%)	-
46-60	25 (24.0%)	10 (22.72%)	15 (25.0%)	-
>60	27 (25.96%)	11 (25.0%)	16 (26.66%)	-

Fosfomycin susceptibility among urinary tract pathogens

Of the 407 UTI patients, 353 (86.73%) were susceptible to fosfomycin and 54 (13.26%) were resistant. The two primary causative organisms were *E. coli *(303; 74.44%) and *K. pneumoniae *(104; 25.55%), showing a predominantly *E. coli*-driven cohort. Comparison of demographic variables between the susceptible and resistant groups showed no statistically significant differences in age (p > 0.005) or gender distribution (p > 0.005). Similarly, stratification by age group demonstrated no significant association with resistance pattern (p > 0.005). These results collectively indicate that age and gender are not significant independent determinants of fosfomycin resistance in this group. However, bacterial species demonstrated a highly significant association with fosfomycin resistance (p < 0.005). Among *K. pneumoniae *isolates, 53 (50.96%) out of 104 were resistant to fosfomycin, accounting for 53 (98.14%) of the 54 total resistant isolates. In comparison, only one (0.33%) of 303 *E. coli *isolates was resistant, accounting for 1 (1.85%) of the 54 total resistant isolates (Table 4).

**Table 4:** Patient characteristics by fosfomycin susceptibility. Data are presented as mean ± SD/median (Q1–Q3) for continuous variables and as n (%) for categorical variables. p-value: Wilcoxon rank sum test; Pearson’s chi-squared test.

Characteristics	Overall, N = 407	Sensitive, N = 353	Resistant, N = 54	χ² (df)	p-value
Age (median)	45	45	47	-	>0.005
IQR	27, 61	26, 61	31, 61		
Sex	-	-	-	-	-
Male	223 (54.79%)	194 (54.95%)	29 (53.70%)	0.0001 (1)	-
Female	184 (45.20%)	159 (45.04%)	25 (46.29%)		>0.005
Organism	-	-	-	-	-
*E. coli*	303 (74.44%)	302 (85.55%)	1 (1.85%)	-	-
				-	<0.001
*K. pneumoniae*	104 (25.55%)	51 (14.44%)	53 (98.14%)	168.1 (1)	-
Age group	-	-	-	-	-
<18	44 (10.81%)	41 (11.61%)	3 (5.55%)	-	-
				-	-
				-	-
18–40	130 (31.94%)	111 (31.44%)	19 (35.18%)	-	>0.005
41–60	130 (31.94%)	112 (31.72%)	18 (33.33%)	-	-
>60	103 (25.30%)	89 (25.21%)	14 (25.92%)	1.84 (3)	-

Logistic regression and ROC curve

**Figure 3:**
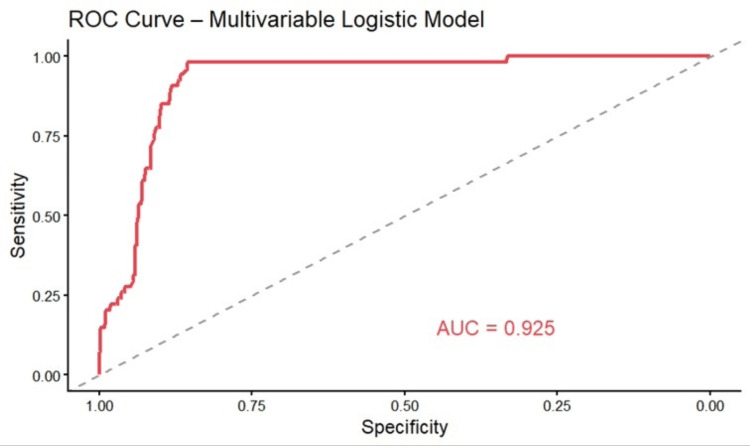
ROC curve of the multivariable logistic regression model (age, sex, organism) predicting Fosfomycin resistance. The red curve plots sensitivity against specificity; the grey dashed line denotes chance-level discrimination (AUC = 0.5). The model demonstrated outstanding discriminative performance (AUC = 0.925), correctly classifying resistant and susceptible isolates in 92.5% of cases. AUC, area under the curve; ROC, receiver operating characteristic.

Logistic regression analysis identified bacterial species as the sole statistically significant independent predictor of fosfomycin resistance. *K. pneumoniae *was associated with a significantly elevated risk of resistance compared to *E. coli *(OR = 322; 95% CI: 68.0-5765; p < 0.001) (Table 5), indicating that *K. pneumoniae *isolates were over 300 times more likely to be resistant to fosfomycin than *E. coli *isolates after adjusting for age and sex. In contrast, neither age (OR = 1.01; 95% CI: 0.99-1.03; p = 0.3) nor sex (female vs. male: OR = 1.01; 95% CI: 0.47-2.16; p > 0.9) was significantly associated with resistance, confirming that host demographic factors do not influence fosfomycin susceptibility in this group. The model displayed excellent performance in differentiating cases, with an area under the ROC curve (AUC = 0.925), indicating that the model, driven primarily by organism type, correctly distinguished resistant from susceptible isolates in 92.5% of cases, well above the threshold for excellent discrimination (AUC > 0.8) (Figure 3).

**Table 5:** Logistic regression: predictors of fosfomycin resistance. CI: confidence interval, OR: odds ratio.

Characteristics	OR	95% CI	p-value
Age	1.01	0.99, 1.03	0.3
Sex	-	-	-
Male	-	-	-
Female	1.01	0.47, 2.16	>0.9
Organism	-	-	-
*E. coli*	-	-	-
*K. pneumoniae*	322	68.0, 5,765	<0.001

## Discussion

The present study assessed fosfomycin susceptibility patterns among 407 UTI patients at AIIMS, Rishikesh, a tertiary care centre in northern India, providing local epidemiological surveillance data for two predominant uropathogens, *E. coli* and *K. pneumoniae*. The overall group was mostly male (55.4%), with a median age of 47 years, and *E. coli* was the most frequently isolated organism (69%), consistent with global epidemiological reports in which *E. coli* accounts for 70%-85% of all community-acquired UTIs [3]. The present analysis identified that *E. coli *was susceptible to fosfomycin (99.7%), except for one isolate, whereas *K. pneumoniae* showed high resistance (51.0%), along with other bacterial species causing UTI. Patient age and gender were not independent factors for resistance. The fosfomycin susceptibility rate of 99.7% observed in *E. coli *in the present study is consistent with, and at the higher end of, published data from India and other regions. Studies from northern Haryana state reported 95% susceptibility among ESBL-producing E.coli and 96% among non-ESBL producers [4], while a multi-centre European study (SURF study) documented 96.4% fosfomycin susceptibility in *E.coli* urinary isolates, observing that fosfomycin remained active against >90% of cephalosporin-resistant strains [11]. Similarly, a study from Rajasthan, India (July 2023 to June 2024), confirmed fosfomycin and nitrofurantoin as preferred empirical agents for MDR *E. coli* UTIs in both outpatient and inpatient settings [12]. These findings provide reassurance regarding the cohort's near-complete susceptibility, affirming oral fosfomycin's ongoing value as an initial therapy for uncomplicated *E. coli* UTIs locally, where resistance to ciprofloxacin, trimethoprim/sulfamethoxazole, and ampicillin is gradually increasing.

Analysis of ESBL status unravelled a significant sex-based disparity in *E. coli* infections: males were significantly more represented in the ESBL-positive group (71% vs. 29%; p < 0.001), while no such association was observed in *K. pneumoniae* (p > 0.999). This finding may reflect the higher prevalence of healthcare-associated and complicated UTIs in males, including catheter-associated infections and urological co-morbidities, which are known to select for ESBL-producing strains due to prior antibiotic exposure. In contrast, *K. pneumoniae* ESBL status was not significantly influenced by age, sex, or any demographic variable, suggesting that ESBL acquisition in this organism may be driven more by hospital-level selective pressure or clonal transmission than by individual patient-level risk factors.

The most clinically significant finding of the present study was the elevated fosfomycin resistance rate of 51.0% among *K. pneumoniae* isolates, with logistic regression confirming that *K. pneumoniae* was over 300 times more likely to be fosfomycin-resistant than *E. coli* after adjusting for age and sex (OR = 322; 95% CI: 68.0-5765; p < 0.001). This figure is considerably higher than resistance rates reported in some previous Indian studies; for instance, a study from Christian Medical College, Vellore, reported 94% susceptibility among carbapenem-resistant *Klebsiella *spp. UTI isolates [13], and a retrospective UK cohort documented 19% fosfomycin resistance in *Klebsiella* spp. [14]. The substantially higher resistance rates observed in the present study likely reflect the tertiary referral nature of the study site, which attracts patients with complex, refractory, and healthcare-associated infections disproportionately caused by MDR *K. pneumoniae* strains. The chromosomally encoded fosA glutathione transferase gene, present in the majority of *K. pneumoniae* strains, is the primary molecular mechanism underlying this intrinsic resistance, and its expression is further amplified under antibiotic selection pressure in hospital environments [6,7].

It is known that current CLSI M100 guidelines (2024 edition) [8] do not provide validated interpretive breakpoints for oral fosfomycin against *K. pneumoniae*, and the IDSA 2024 AMR Guidance [9] advises against fosfomycin use for *K. pneumoniae* UTI given the risk of clinical failure driven by the fosA gene. However, the pattern of fosfomycin susceptibility in *K. pneumoniae* was included in this study because *K. pneumoniae* constituted 25.6% of uropathogens in this study and is globally recognised as the second most common uropathogen. The escalating prevalence of MDR and carbapenem-resistant *K. pneumoniae* (CRE/KPC) has positioned fosfomycin as one of the very few remaining oral antibiotic options in resistant infections, with clinical cure demonstrated in selected cases using high-dose intravenous fosfomycin [15]. In vitro studies have demonstrated relevant fosfomycin activity against MDR *K. pneumoniae* under conditions that simulate the urinary environment [5]. Fosfomycin testing against *K. pneumoniae* using *E. coli* breakpoints as a pragmatic surrogate is an accepted research approach used across multiple published studies from South Africa, South Korea, Iran, and India [5,14]. Critically, the high resistance rate documented here (51.0%) itself constitutes a policy-relevant finding, precisely the type of local epidemiological evidence required to support and customise guideline recommendations that advise against fosfomycin use in *K. pneumoniae* UTI, and to guide clinicians in this region to avoid empirical fosfomycin prescribing when *K. pneumoniae* infection is suspected.

Fosfomycin resistance status was not substantially correlated with patient age or biological sex in this investigation, which is in line with findings from other published series [14]. The age distribution of resistant isolates was broadly uniform across all age groups (5.6%-35%), and the sex ratio was virtually identical between susceptible and resistant groups (OR = 1.01; p > 0.9). This finding has practical clinical implications: it suggests that clinician decision-making regarding fosfomycin use should be driven by organism identification and susceptibility testing rather than patient demographics, and that no specific age group or sex requires heightened empirical precaution on demographic grounds alone.

The multivariable logistic regression model demonstrated outstanding discriminative performance (AUC = 0.925), driven strongly by the organism variable. This high AUC reflects the near-binary nature of the fosfomycin resistance distribution in this dataset, with almost all resistance concentrated in *K. pneumoniae* rather than complex multivariable interactions, and should be interpreted in this context. The model was well calibrated, as confirmed by the Hosmer-Lemeshow goodness-of-fit test and bootstrap calibration curve (B = 200). A nomogram was constructed to allow individualised probability estimation; however, given the dominant influence of organism type, its principal clinical value lies in formally quantifying resistance risk in patients from whom *K. pneumoniae* is isolated, rather than providing nuanced demographic risk stratification.

Limitations

The retrospective single-centre design at a tertiary referral hospital limits the generalisability of resistance rates to primary and community care settings, where resistance prevalence may differ substantially. Also, the application of *E. coli *CLSI breakpoints to *K. pneumoniae* isolates in the absence of validated species-specific criteria introduces interpretive uncertainty, and the results should be considered in vitro phenotypic data rather than clinically actionable susceptibility categories. Molecular characterisation of resistance mechanisms (e.g., fosA, fosA3, plasmid-mediated genes) was beyond the scope of this study due to limited resources; future work incorporating whole-genome sequencing would provide mechanistic insight into the high resistance prevalence observed. Clinical outcome data were not collected, precluding direct correlation between in vitro susceptibility and treatment success.

## Conclusions

Fosfomycin remains a clinically reliable oral drug option for uncomplicated UTIs caused by *E. coli*, and its efficacy is unlikely to be undermined by demographic or institutional factors in the northern India setting. However, its role is organism-specific and should not be generalised comprehensively across uropathogens. The clear separation in fosfomycin susceptibility between *E. coli *and *K. pneumoniae *reinforces a broader principle that empirical antibiotic selection, however evidence-based, carries an essential risk when applied to pathogens with heterogeneous and unpredictable resistance profiles. Culture-guided therapy is not merely a microbiological formality but a clinical necessity when *K. pneumoniae *is suspected or isolated. These findings support existing guideline conservatism around empirical fosfomycin use for non-*E. coli *pathogens and highlight the continuing importance of local antimicrobial stewardship grounded in institutional susceptibility data.
